# A non-tight junction function of claudin-7—Interaction with integrin signaling in suppressing lung cancer cell proliferation and detachment

**DOI:** 10.1186/s12943-015-0387-0

**Published:** 2015-06-17

**Authors:** Zhe Lu, Do Hyung Kim, Junming Fan, Qun Lu, Kathryn Verbanac, Lei Ding, Randall Renegar, Yan-Hua Chen

**Affiliations:** School of Medicine, Hangzhou Normal University, Hangzhou, 310036 China; Department of Anatomy and Cell Biology, Brody School of Medicine, East Carolina University, Greenville, NC 27834 USA; Leo Jenkins Cancer Center, Brody School of Medicine, East Carolina University, Greenville, NC 27834 USA; Department of Surgery, Brody School of Medicine, East Carolina University, Greenville, NC 27834 USA

**Keywords:** Claudin-7, Integrin β1, Cell proliferation, Cell-matrix interactions, Lung cancer cells

## Abstract

**Background:**

Claudins are a family of tight junction (TJ) membrane proteins involved in a broad spectrum of human diseases including cancer. Claudin-7 is a unique TJ membrane protein in that it has a strong basolateral membrane distribution in epithelial cells and in tissues. Therefore, this study aims to investigate the functional significance of this non-TJ localization of claudin-7 in human lung cancer cells.

**Methods:**

Claudin-7 expression was suppressed or deleted by lentivirus shRNA or by targeted-gene deletion. Cell cycle analysis and antibody blocking methods were employed to assay cell proliferation and cell attachment, respectively. Electron microscopy and transepthelial electrical resistance measurement were performed to examine the TJ ultrastructure and barrier function. Co-immunolocalization and co-immunoprecipitation was used to study claudin-7 interaction with integrin β1. Tumor growth *in vivo* were analyzed using athymic nude mice.

**Results:**

Claudin-7 co-localizes and forms a stable complex with integrin β1. Both suppressing claudin-7 expression by lentivirus shRNA in human lung cancer cells (KD cells) and deletion of claudin-7 in mouse lungs lead to the reduction in integrin β1 and phospho-FAK levels. Suppressing claudin-7 expression increases cell growth and cell cycle progression. More significantly, claudin-7 KD cells have severe defects in cell-matrix interactions and adhere poorly to culture plates with a remarkably reduced integrin β1 expression. When cultured on uncoated glass coverslips, claudin-7 KD cells grow on top of each other and form spheroids while the control cells adhere well and grow as a monolayer. Reintroducing claudin-7 reduces cell proliferation, upregulates integrin β1 expression and increases cell-matrix adhesion. Integrin β1 transfection partially rescues the cell attachment defect. When inoculated into nude mice, claudin-7 KD cells produced significantly larger tumors than control cells.

**Conclusion:**

In this study, we identified a previously unrecognized function of claudin-7 in regulating cell proliferation and maintaining epithelial cell attachment through engaging integrin β1.

**Electronic supplementary material:**

The online version of this article (doi:10.1186/s12943-015-0387-0) contains supplementary material, which is available to authorized users.

## Background

Lung cancer is the leading cause of cancer death for both men and women in the United States and nearly 85 % of patients who develop lung cancer die from it. The overall 5-year survival rate of lung cancer is as low as 15 % [1]. Lung cancer usually forms in the cells lining air passages. The vast majority of lung cancers are carcinomas-malignancies that arise from epithelial cells. Tight junctions (TJs) are the most apical component of the junctional complex and provide the critical support of cell-cell adhesion in epithelial cells [2]. The disruption of cell adhesion promotes cancer progression, invasion and metastasis [3, 4]. TJs serve as a barrier regulating the ions and small molecules through the paracellular pathway [5]. Both downregulation and upregulation of TJ proteins can alter the organized arrangement of TJ strands, allowing more nutrients and growth factors to cancerous cells, thus acquiring the cells with invasive behavior [6].

Claudins are tetraspan proteins with molecular weights of 20–27 kDa [7]. They are the major structural and functional components of TJs. Abnormal expressions and mislocalization of claudins are frequently observed in epithelial-derived cancers [8–11]. Moldvay *et al.* have analyzed the expression profile of different claudins in lung cancers and found that claudin-7 is downregulated in several types of lung cancers including the squamous cell carcinoma at the mRNA level [12]. Our previous study demonstrates that claudin-7 is strongly expressed in benign bronchial epithelial cells with a predominant cell-cell junction staining pattern while it is either altered with discontinued weak expression or completely absent in lung cancers [13]. However, the exact roles of claudin-7 in lung tumorigenesis are largely unknown.

Although claudins are well-known apical TJ proteins, recent antibody-based studies indicated that several claudins, including claudin-7, are not only localized at the apical TJs but also have a strong basolateral membrane distribution in the epithelia of various tissues [14–16]. These observations suggest that claudins could be involved in cell-matrix interactions. The principal proteins at the basolateral membrane responsible for anchoring cells to extracellular matrix proteins are integrins [17]. Integrins are heterodimers with α and β subunits and play essential roles in cell attachment, survival, migration and invasion [18, 19].

In this study, we identified that claudin-7 co-localized and formed a protein complex with integrin β1 in human lung cancer cells. Suppression of claudin-7 not only promoted cell proliferation, but also disrupted the localization and downregulated the expression of integrin β1 at both mRNA and protein levels, resulting in the severe defective cell attachment. Introducing integrin β1 into claudin-7-deprived cells partially rescued the defect in cell attachment. Thus, claudin-7 exhibits a non-TJ function in regulating cell attachment through integrin β1.

## Results

### Increased cell proliferation and cell cycle progression in claudin-7 KD cells

**Fig. 1:**
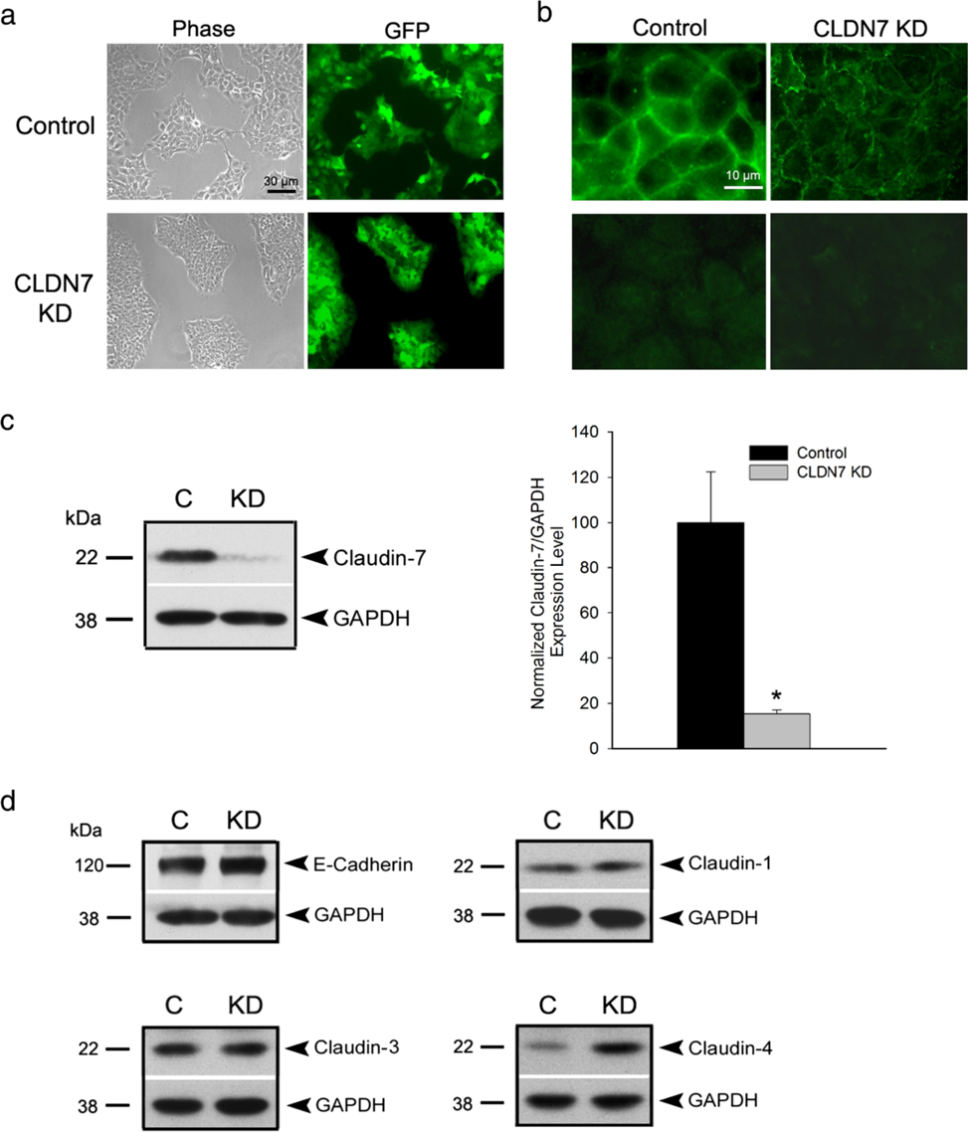
Knockdown of claudin-7 in HCC827 lung cancer cells. **a** Representative phase and green fluorescence images of live control and claudin-7 KD cells. Both control and claudin-7 shRNA lentivirus constructs contain a GFP expression sequence. **b** Top panel is the anti-claudin-7 immunofluorescence staining of control and claudin-7 KD cells. Claudin-7 level was dramatically decreased in claudin-7 KD cells. The bottom panel is the anti-GFP immunofluorescence staining. The cells were fixed with 100 % methanol, which lead to GFP proteins leaking out of the cells so that the green fluorescence shown in the top panel was only the claudin-7 signal. **c** Western blot shows the diminished level of claudin-7 in the KD cells (*left*). The statistical analysis of claudin-7 expression in control and KD cells from five independent experiments is shown on right. *P < 0.05. **d** Protein expression levels of E-cadherin, claudin-1, −3, and −4 in control and KD cells. Only claudin-4 expression level was increased in KD cells. GAPDH served as a loading control

Our results revealed that HCC827 claudin-7 KD cells became smaller in size, less spread out, and grew in an isolated patch pattern while the control cells were spread out and uniformly distributed over the plate (Fig. 1a). Claudin-7 immunofluorescence staining (Fig. 1b) and western blot (Fig. 1c) showed the successful knockdown of claudin-7 using #2 shRNA vector against claudin-7. HCC827 cells infected with #3 shRNA vector against claudin-7 are shown in Additional file 1: Figure S1 and these claudin-7 knockdown cells were designated as KD2 cells. Expression level of claudin-4 was increased in claudin-7 KD cells (Fig. 1d). There were no changes of expression levels of adherens junction protein E-cadherin and TJ proteins claudin-1 and −3 after claudin-7 was knocked down (Fig. 1d). There was no expression of claudin-2 in HCC827 lung cancer cells (data not shown). We have also knocked down claudin-7 expression in NCI-H358 (H358) human lung cancer cells (Additional file 2: Figure S2).

**Fig. 2:**
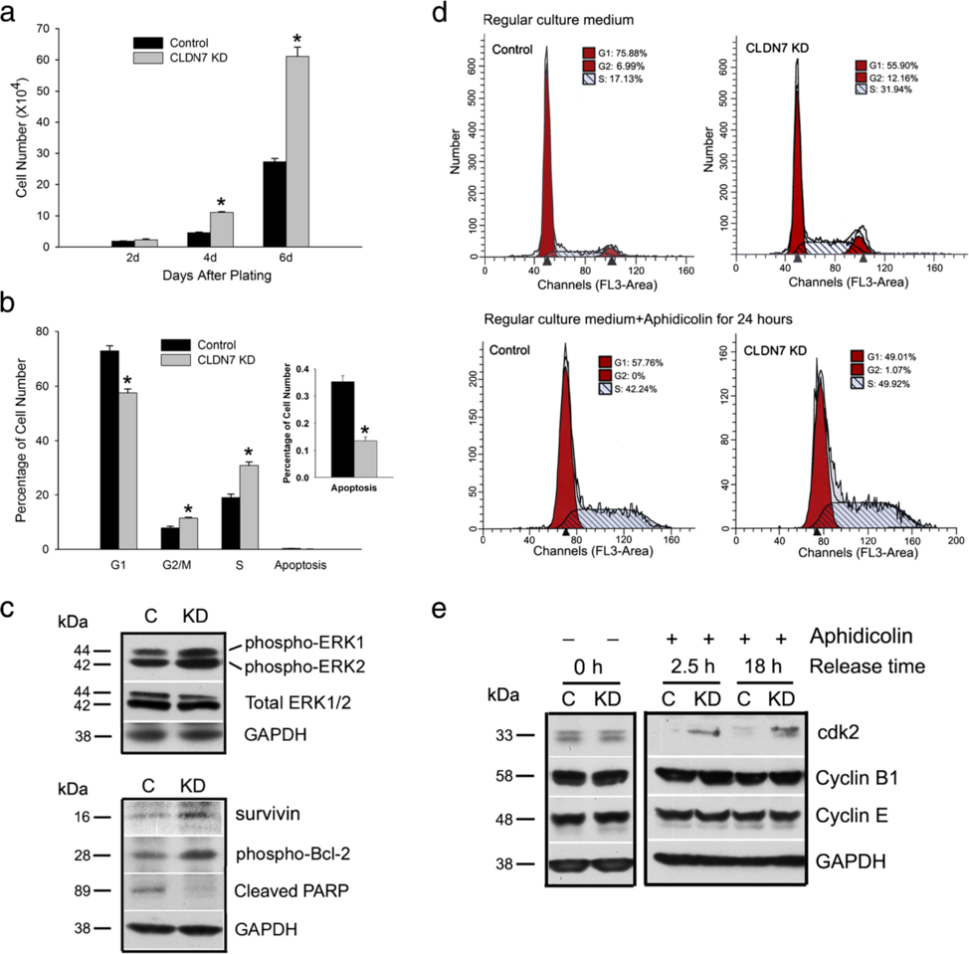
Increased cell proliferation in claudin-7 KD cells. **a** Five × 10^3^ control and claudin-7 KD cells were seeded into 24-well plates and the cell number was counted for each sample on days 2, 4, and 6 after plating. Claudin-7 KD cells show significantly higher proliferation rate compared to the control cells on days 4 and 6. *P < 0.05. **b** Three days after plating, cells were trypsinized, centrifuged, and stained with propidium iodide and subjected to cell cycle analysis by flow cytometry. Claudin-7 KD cells show a higher percentage of G2/M and S phase cells and lower percentage of apoptotic cells. *P < 0.05. **c** Representative western blots show increased levels of phospho-ERK1/2, survivin, and phospho-Bcl-2 and decreased level of cleaved PARP in claudin-7 KD cells when compared to control cells. A total of 30 μg proteins were loaded for each sample. **d** The top panel is the representative cell cycle analysis of control and claudin-7 KD cells in regular culture medium 3 days after plating. The bottom panel was taken after 24 h of aphidicolin treatment, showing that both cell lines were inhibited at S phase. **e** Control and KD cells were treated without (*left*) and with 24 h of aphidicolin (*right*). Cell lysates were collected 2.5 and 18 h after releasing from aphidicolin treatment

Because claudin-7 KD cells were smaller in size with high density in each patch, we performed a cell number count experiment to investigate the cell growth rates in both control and claudin-7 KD cells. We found that claudin-7 expression was associated with decreased cell proliferation. After claudin-7 was knocked down in HCC827 cells, the cell proliferation rate increased. Starting from day 4, the number of claudin-7 KD cells was twice more than that of control cells (Fig. 2a). Similarly, the claudin-7 KD2 cells (infected with #3 shRNA vector against claudin-7) also showed a significantly increased cell proliferation for all three days examined compared to the control cells (Additional file 3: Figure S3A). The cell doubling time for control and claudin-7 KD cells were 24 and 12 h, respectively. Cell cycle analysis revealed that a higher percentage of claudin-7 KD cells was in the DNA synthesis phase (S phase) and mitosis (G2/M) phase (Fig. 2b), consistent with the higher growth rate observed in the claudin-7 KD group.

We proceeded to examine several proteins involved in cell proliferation, cell survival and apoptosis by western blot. Our results revealed increased levels of phospho-ERK1/2, phospho-Bcl-2 and survivin and decreased level of cleaved PARP in claudin-7 KD cells (Fig. 2c). The claudin-7 KD2 cells also displayed an increased level of phospho-ERK1/2 and a decreased level of cleaved PARP as shown in Additional file 3: Figure S3B. Similar results were obtained in H358 claudin-7 KD cells in that these cells were higher in cell proliferation and had increased phospho-ERK1/2 level and decreased cleaved PARP level (Additional file 4: Figure S4).

Figure 2d shows the cell cycle analysis before and after 24 h of aphidicolin treatment. Aphidicolin blocks cells in S phase and prevents G1 cells from entering mitosis. Cyclin dependent kinase 2 (Cdk2) is required for G1 to S phase transition [20]. After 2.5 and 18 h of releasing the cells from aphidicolin, the cdk2 expression levels were higher in claudin-7 KD cells (Fig. 2e), indicating that more KD cells were transitioning from G1 to S phase. There were no significant differences in expression levels of cyclin B1 and cyclin E between control and claudin-7 KD cells.

We used TUNEL (Terminal deoxynucleotidyl transferase dUTP nick end labeling) staining method to detect the apoptosis. Although the total population of apoptotic cells in both control and claudin-7 KD cells was low, the claudin-7 KD cells showed a lower percentage of apoptotic cells than the control cells as shown in Additional file 5: Figure S5.

### Impaired cell attachment and decreased integrin β1 expression in claudin-7 KD cells

**Fig. 3:**
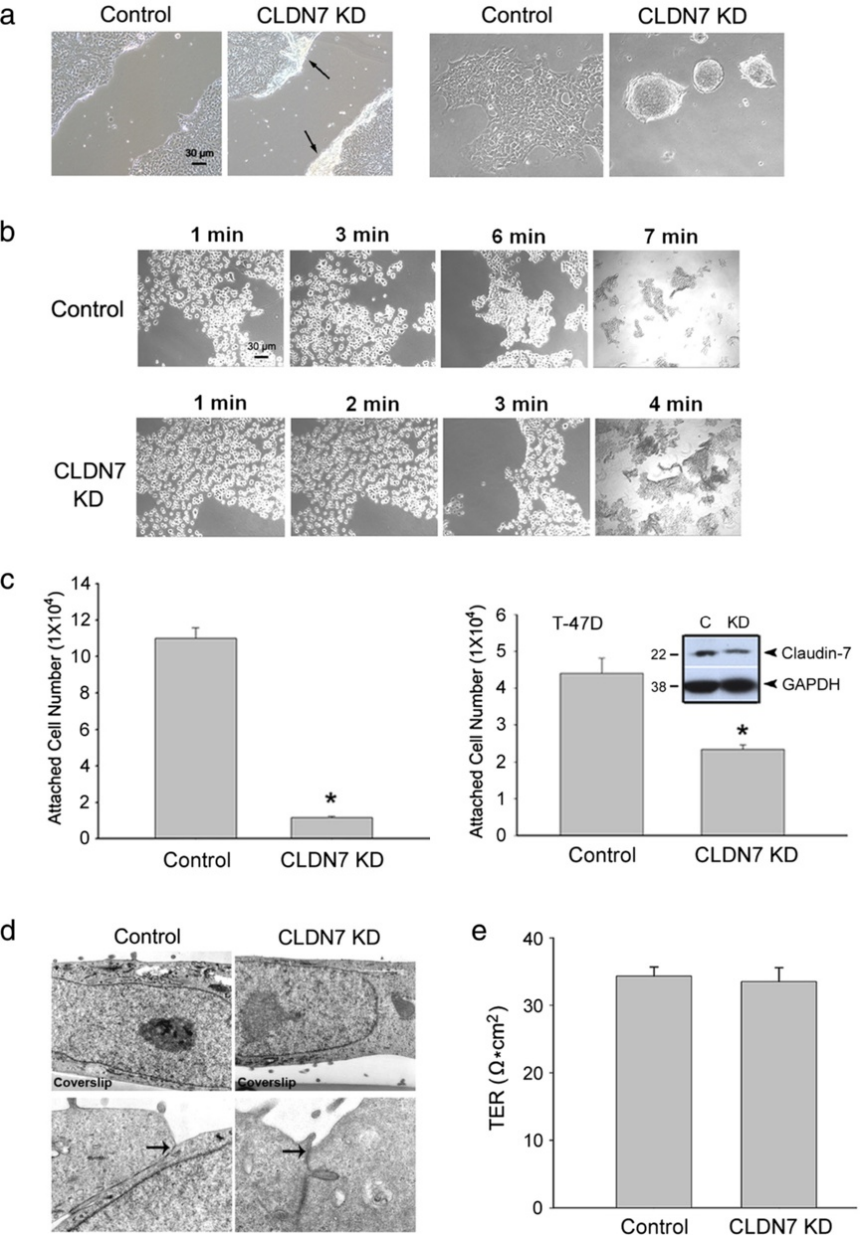
Reduced cell-matrix adhesion in claudin-7 KD cells. **a** *Left panel*: Scratches were made on the confluent control and claudin-7 KD cell monolayer. Claudin-7 KD cells were easily peeled off along the scratch as shown in arrows, while the control cells were well attached to the plate. *Right panel*: When cultured on uncoated glass coverslips, claudin-7 KD formed spheroids while the control cells were able to spread out and formed a monolayer. **b** Monolayer cultures of control and claudin-7 KD cells were exposed to trypsinization and phase images were taken every minute. Claudin-7 KD cells took less time (4 min) to be fully lifted by trypsin than the control cells (7 min). **c** Cell attachment assay. Two × 10^5^ control and KD cells were plated to each well of the collagen IV-coated 24-well plates. Four hours later, the unattached cells were washed off and the attached cells were trypsinized and counted. KD cells showed significantly reduced cell attachment compared to the control cells (*left*). The significantly reduced cell attachment was also observed in T-47D breast cancer cells with claudin-7 knockdown by the same shRNA approach (*right*). *P < 0.05. The insert shows the knockdown level of claudin-7 in T-47D cells. **d** Electron microscopy shows a larger gap between claudin-7 KD cells and the coverslip than that between control cells and the coverslip. TJs in both cell lines were largely intact (arrows). Magnifications: top, ×25,000; bottom, ×50,000. **e** TER measurement indicates that there was no significant change in the barrier function of TJs after claudin-7 was knocked down

The wound healing assay was performed to examine the migration ability of both control and claudin-7 KD cells. We found that claudin-7 KD cells were peeled off easily along the edge of the wound each time after making a scratch to the monolayer (Fig. 3a, left). When the cells were plated on the uncoated coverslips, control cells adhered well and formed a monolayer on the coverslip while the KD cells grew on top of each other and formed spheroids (Fig. 3a, right). In addition, it took KD cells about 40 % less time to be completely lifted from the culture dish by trypsin than control cells (Fig. 3b). Cell attachment assay also revealed that only about 10 % KD cells were attached to the culture plate compared to the control cells four hours after plating (Fig. 3c, left). Similar results were also obtained in claudin-7 KD2 cells (Additional file 6: Figure S6A). The attached cell number was significantly reduced in claudin-7 KD2 cells compared to that of the control cells. In addition, lentivirus claudin-7 shRNA was used to knockdown claudin-7 expression in breast cancer cell line T-47D (Fig. 3c, right). Only 45 % T-47D claudin-7 KD cells were attached to the plate compared to the control cells four hours after plating. Electron microscopy revealed larger gaps between HCC827 claudin-7 KD cells and coverslip than that between control cells and coverslip while TJs were shown largely intact in both cell lines under high magnification (Fig. 3d, arrow). Transepithelial resistance (TER) measurement revealed that suppression of claudin-7 in HCC827 cells did not affect the barrier function of TJs (Fig. 3e). These results indicate that cell attachment machinery, not functions of TJs, was impaired in claudin-7 KD cells.

**Fig. 4:**
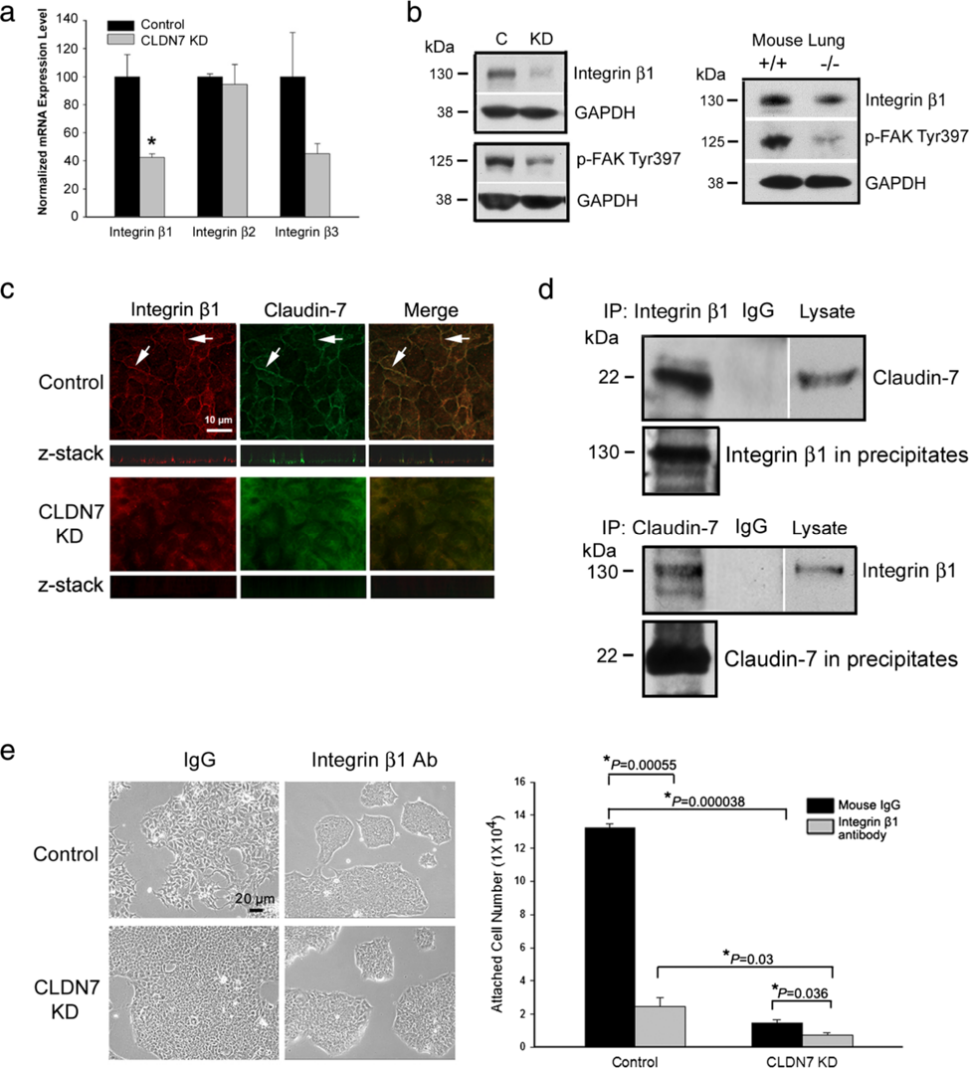
Decreased mRNA and protein levels and disrupted localization of integrin β1 in claudin-7 KD cells. **a** Real-time RT-PCR was performed on integrins β1, β2, and β3. The mRNA level of integrin β1 was significantly reduced in claudin-7 KD cells. *P < 0.05. **b** Western blots show that integrin β1 and phospho-FAK levels were decreased in both claudin-7 KD cells (*left*) and claudin-7 knockout mouse lungs (*right*). At least six independent experiments were performed. **c** Immunofluorescence staining shows the localization of claudin-7 and integrin β1 in control and KD cells. Z-stack shows the co-localization of claudin-7 with integrin β1 at the basolateral surface in the merged image (*top*). In contrast, the localization of integrin β1 was greatly disrupted in the claudin-7 KD cells; it was no longer at the cell membrane (*bottom*). Arrows in control cells indicate the partial co-localization of claudin-7 with integrin β1. **d** Claudin-7 co-immunoprecipitated with integrin β1. Control cells were lysed in RIPA buffer without SDS and immunoprecipitated with either integrin β1 or claudin-7 antibody and then probed with either claudin-7 or integrin β1. **e** Control and KD cells were treated with anti-integrin β1 adhesion-blocking antibody or mouse IgG as a control. Cells were treated with the antibodies for two days, then cells were trypsinized and replated. The unattached cells were washed away two hours after replating, and the cells attached to the culture plate were trypsinized and counted. *P* values were shown above the bars on the right

Defective cell attachment in claudin-7 KD cells indicates a non-TJ function of claudin-7 in cell-matrix adhesions. Integrins are the principal receptors responsible for binding the cells to extracellular matrix. After screening for major subunits, we found that integrin β1 was greatly decreased at both mRNA and protein levels (Fig. 4a and b). In addition, the phosphorylation level of focal adhesion kinase (FAK) was also greatly reduced in claudin-7 KD cells (Fig. 4b, left). The decreased expression levels of integrin β1 and phospho-FAK were observed in claudin-7 KD2 cells as well (Additional file 6: Figure S6B). Moreover, the reduced signals for integrin β1 and phospho-FAK were confirmed in the claudin-7 knockout mouse lungs (Fig. 4b, right). Immunofluorescence staining showed a strong basolateral staining of claudin-7 (Fig. 4c, top, Z-stack) that was co-localized with integrin β1. Knockdown of claudin-7 disrupted integrin β1 localization in that it was no longer at the cell membrane (Fig. 4c, bottom). Single staining of integrin β1 or claudin-7 as well as anti-secondary antibody only were shown in Additional file 7: Figure S7. Co-immunoprecipitation confirmed that claudin-7 and integrin β1 formed a protein complex in HCC827 cells (Fig. 4d). The decreased level of phospho-FAK in claudin-7 KD cells shown in Fig. 4b was consistent with the phenotype observed in claudin-7 KD cells in that they were less spread out than control cells (Fig. 1a).

To determine whether integrin β1 is the key regulator mediating cell-matrix adhesions, we used mouse anti-human integrin β1 adhesion-blocking antibody to treat both control and claudin-7 KD cells. Mouse IgG treatment was used as a negative control (Fig. 4e). Anti-integrin β1 antibody treatment significantly interfered with the cell attachment in both control and KD cells when compared to the mouse IgG treatment (Fig. 4e, right). The cell attachment assay revealed that the attached cells were significantly reduced in both control and claudin-7 KD cells after integrin β1 antibody treatment. This adhesion-blocking experiment indicates that integrin β1 is one of the key regulators in mediating cell attachment in HCC827 cells.

Importantly, H358 claudin-7 KD cells also formed spheroids when they were plated on the uncoated coverslips while control cells adhered well (Additional file 8: Figure S8A). Results from cell attachment assay indicated that only about 15 % H358 claudin-7 KD cells were attached to the culture plate when compared to the control cells (Additional file 8: Figure S8B). The expression levels of integrin β1 and phospho-FAK were also reduced in H358 claudin-7 KD cells (Additional file 9: Figure S9A). Immunofluorescent staining showed the partial co-localization of claudin-7 with integrin β1 (Additional file 9: Figure S9B). In addition, claudin-7 interacted with integrin β1 and formed a protein complex in H358 cells as well (Additional file 9: Figure S9C).

### Rescue claudin-7 KD cell defects by exogenous claudin-7 or integrin β1 expression

**Fig. 5:**
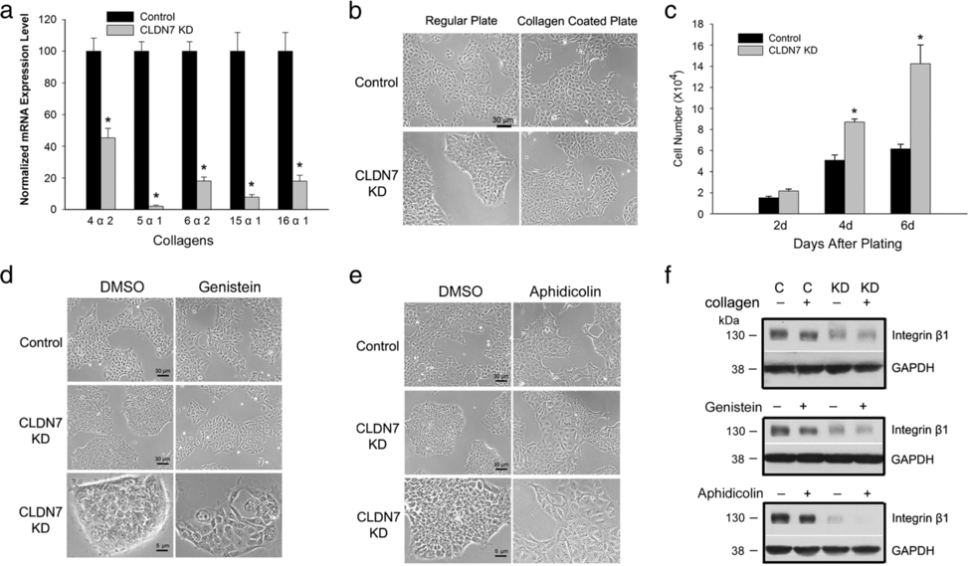
Treatments of claudin-7 KD cells with collagens, genistein and aphidicolin. **a** Real-time RT-PCR revealed that several collagens were significantly decreased in claudin-7 KD cells when compared to the control cells. *P < 0.05. **b** By culturing the cells on type IV collagen-coated culture plates, claudin-7 KD cells displayed the increased cell size and were more spread out. **c** Five × 10^3^ control or claudin-7 KD cells were plated on type IV collagen-coated culture plates. Cell number was counted on days 2, 4, and 6 after plating. Claudin-7 KD cells showed a higher proliferation rate than control cells. *P < 0.05. **d** Phase images of control and KD cells after 24 h of DMSO (control) or 200 μM genistein treatment. **e** Phase images of control and KD cells after 24 h of DMSO or 5 μg/ml aphidicolin treatment. **f** Control and KD cells cultured on the collagen IV coated plate, or treated with genistein or aphidicolin were lysed, loaded onto SDS-polyacrylamide gel, and probed with anti-integrin β1 antibody

Cell-matrix adhesions involve not only cells and cell-matrix adhesion proteins such as integrin β1, but also involve extracellular matrix proteins. The most abundant extracellular proteins are collagens. Real-time RT-PCR revealed that collagen subunits 4α2, 5α1, 6α2, 15α1, and 16α2 were all significantly decreased at the transcriptional levels in claudin-7 KD cells (Fig. 5a). Thus, we cultured the claudin-7 KD cells on type IV collagen-coated plates to see whether a deficient collagen deposition was the primary consequence of claudin-7 suppression and whether type IV collagen was able to rescue the defects of claudin-7 KD cells. We found that claudin-7 KD cells grown on type IV collagen-coated plates became larger in size, more spread out (Fig. 5b), and showed partially improved cell attachment as determined by the prolonged trypsinization time when compared to the KD cells cultured on regular culture plates (data not shown). However, the cell proliferation rate was still significantly higher in claudin-7 KD cells when compared to control cells (Fig. 5c).

To investigate whether slowing down the cell proliferation rate is able to rescue the phenotype of KD cells, we treated control and claudin-7 KD cells with genistein or aphidicolin for 24 h. Our results showed that claudin-7 KD cells responded to both drug treatments in that the cell size became enlarged when compared to untreated cells (Fig. 5d&e). However, culturing KD cells on type IV collagen-coated plates or treating KD cells with genistein or aphidicolin did not increase integrin β1 protein expression level in claudin-7 KD cells (Fig. 5f).

**Fig. 6:**
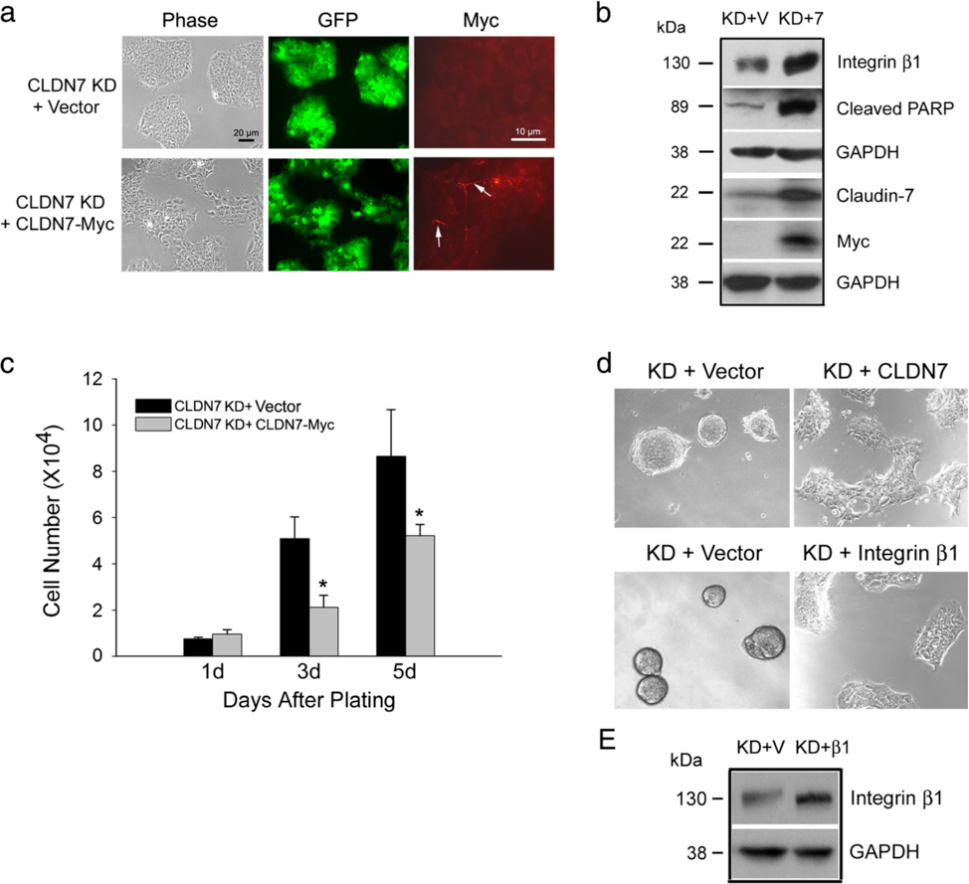
Rescued claudin-7 KD cell defects by exogenous expression of claudin-7 or integrin β1 in KD cells. **a** The *claudin-7* construct tagged with myc was transfected into the HCC827 claudin-7 KD cells. The transfected claudin-7 sequence has three nucleotide differences from lentivirus claudin-7 shRNA (due to mouse and human *claudin-7* difference), so it is resistant to the lentivirus shRNA. The claudin-7 KD cells with *claudin-7-myc* transfection became larger in size and more spread out (phase images). Anti-myc immunofluorescence staining indicated by arrows shows the exogenous claudin-7 expression in claudin-7 KD cells. **b** Lysates from claudin-7 KD cells transfected with vector (KD + V) or *claudin-7*-*myc* (KD + 7) were collected and subjected to western blot analysis. The expression levels of integrin β1 and cleaved PARP were both increased in claudin-7 transfected KD cells. The membrane probed with anti-myc antibody shows the exogenous claudin-7 expression in KD cells. **c** Five × 10^3^ claudin-7 KD cells transfected with vector or *claudin-7*-myc were plated into 24-well plates. Cell number was counted on days 1, 3, 5 after plating. *P < 0.05. **d** Claudin-7 KD cells transfected *claudin-7* (*upper panel*) or *integrin β1* (*lower panel*) construct were trypsinized and plated on uncoated glass coverslips. **e** Lysates from claudin-7 KD cells transfected with vector (KD + V) or *integrin β1* (KD+ β1) were collected and subjected to western blot analysis. The membrane probed with anti-integrin β1 antibody shows the expression level of integrin β1 in KD cells without or with integrin β1 transfection

To confirm that claudin-7 is the key factor in regulating cell proliferation and cell attachment in HCC827 lung cancer cells, we transfected *claudin-7* tagged with myc into the claudin-7 KD cells to investigate whether we can rescue the phenotype of claudin-7 KD cells (Fig. 6a). Exogenous claudin-7-myc expression not only increased integrin β1 and cleaved PARP protein levels (Fig. 6b), but also reversed the morphology observed in claudin-7 KD cells in that they became larger in size, more spread out (Fig. 6a), and showed the significantly slower cell proliferation rate compared to the KD cells without claudin-7 transfection (Fig. 6c). Moreover, after we transfected claudin-7 or integrin β1 back to the KD cells and then plated them on uncoated glass coverslip, claudin-7 or integrin β1 transfected KD cells were adhered well and formed a monolayer while the vector-transfected KD cells still formed spheroid structures (Fig. 6d). These results demonstrate that claudin-7 is critical in regulating cell proliferation and cell attachment and the decreased integrin β1 level is the direct consequence of claudin-7 suppression that is responsible for weakened cell attachment.

### Claudin-7 suppressed tumor growth *in vivo*

**Fig. 7:**
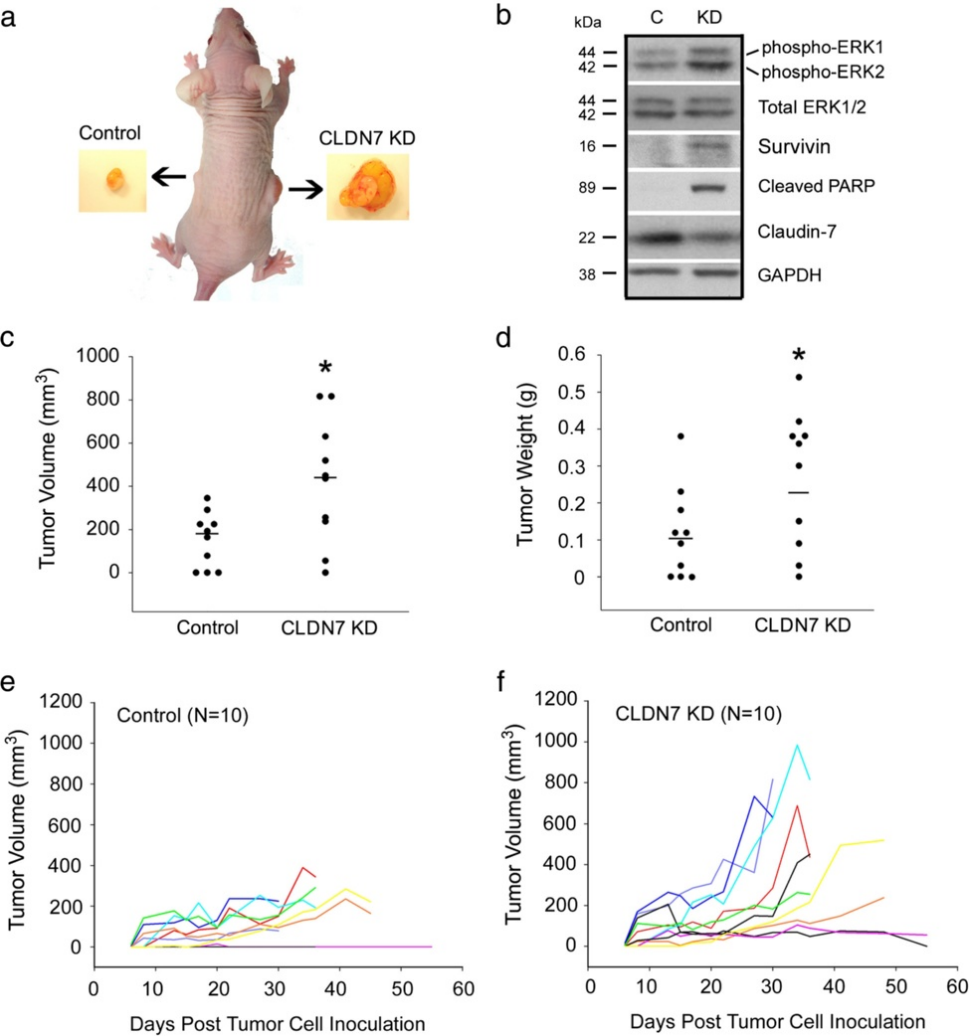
The inhibitory effect of claudin-7 on tumor growth in nude mice. **a** Two × 10^6^ HCC827 control or claudin-7 KD cells were harvested and suspended in the culture medium, then subcutaneously injected into the left and right flanks of a total of 10 nude mice, respectively. Arrows indicate the tumors generated subcutaneously in the representative nude mouse which was sacrificed 30 days post tumor cell inoculation. **b** Western blot analysis shows the increased expression levels of phospho-ERK1/2, survivin, and cleaved PARP in claudin-7 KD cells-induced tumors when compared to those of control cells-induced tumors. **c**, **d** The nude mice were sacrificed and the tumors were removed and measured in both volume and weight. Statistical analysis was conducted on a total of 10 nude mice. Medians were shown as black solid lines in the figures. *P < 0.05. **e**, **f** Growth curves for ten control cells-and ten claudin-7 KD cells-inoculated tumors

To investigate whether claudin-7 can inhibit cell growth *in vivo*, we injected HCC827 control or claudin-7 KD cells into the flanks of the nude mice. As shown in Fig. 7a, the tumor induced by control cells was much smaller than that induced by claudin-7 KD cells. The inserts are the tumors taken out from the left and right sides of the nude mice, representing the control cells-induced and claudin-7 KD cells-induced tumors, respectively. Western blot results showed that claudin-7 KD cells-induced tumors have higher expression levels of phospho-ERK1/2, survivin, and cleaved PARP than those of control cells-induced tumors (Fig. 7b). Fig. 7c and d show the quantitative analysis confirming that the sites inoculated with claudin-7 KD cells developed the tumors significantly larger in size and heavier in weight than those inoculated with control cells, demonstrating that claudin-7 inhibited the tumor growth *in vivo*. Fig. 7e and f show the growth curves of ten control cells-inoculated tumors and ten claudin-7 KD cells-inoculated tumors, respectively. Seven of the ten nude mice inoculated with control cells developed tumors while nine of the ten nude mice inoculated with claudin-7 KD cells developed tumors. Six control tumors grew significantly slower than their paired claudin-7 KD tumors and the slopes of these paired tumor growth curves are significantly different.

## Discussion

In this study, we demonstrated that knockdown of claudin-7 significantly reduced integrin β1 expression and altered its localization in HCC827 and NCI-H358 lung cancer cells. These claudin-7 KD cells displayed the severe cell-matrix adhesion defect that was also observed in breast cancer cell line T-47D. Confocal immunofluorescence microscopy revealed the partial co-localization of claudin-7 with integrin β1 at the basolateral membrane. Co-immunoprecipitation experiments indicated that claudin-7 interacted with integrin β1 and formed a protein complex in the control cells. The cell-matrix adhesion defect can be rescued by transfecting claudin-7 or integrin β1 into claudin-7 KD cells. Our data also demonstrated that knockdown of claudin-7 promoted cell proliferation and propelled cell cycle progression. The *in vivo* experiments indicated that claudin-7 KD cells produced larger and heavier tumors in nude mice when compared to the control cells. It is known that the fast-growing tumor has a higher cell proliferation rate as well as a higher cell apoptosis rate due to lack of nutrition and hypoxia within the tumor tissue [21–23]. In the cell culture system, cancer cells have sufficient nutrition and oxygen. This could explain why a higher expression level of cleaved PARP was observed in Claudin-7 KD cells-induced tumors (Fig. 7b) while claudin-7 KD cells in culture had a decreased level of cleaved PARP (Fig. 2c). Our results suggest that claudin-7 could potentially be a tumor suppressor in lung cancer inhibiting tumor cell growth both *in vitro* and *in vivo*.

Integrins are cell surface receptors that lack intrinsic tyrosine kinase activity. FAK is constitutively associated with integrin β1 of the integrin receptors. The binding of integrin β1 to extracellular matrix proteins leads to the activation of FAK through its auto-phosphorylation at tyrosine 397 site. It has been reported that claudin-2 facilitates the cell-matrix adhesions by increasing the cell surface expression of α2β1 and α5β1-integrin complexes in breast cancer cells, promoting the formation of breast cancer liver metastases [24]. In this study, we found that knockdown of claudin-7 reduced integrin β1 at both mRNA and protein levels. How claudins affect integrin expression is largely unknown. We were unable to detect claudin-7 in the nucleus by immunofluorescence staining, however, we cannot rule out the possibility that claudin-7 translocated into the nucleus at a level that was beyond the immunofluorescence detection sensitivity. Western blot on nuclear extracts is needed to further determine whether claudin-7 is present in the nucleus and can potentially affect integrin β1 transcriptional expression. On the other hand, claudin-7 could indirectly regulate integrin β1 transcription by regulating the transcription factors of integrin β1, such as Pax6 [25], Hox D3 [26], hypoxia-inducible factor (HIF) [27] and c-Myc [28]. One approach to investigate the potential transcriptional factors involved is to apply cDNA microarrays in the future.

Since suppression of claudin-7 not only disrupted cell-matrix adhesions but also caused a higher cell proliferation, we investigated which of these two consequences was the primary effect of claudin-7 knockdown. Frequent cell division can promote cell detachment. During mitosis, cells undergo large morphologic changes; they become detached, form retraction fibers while the cell margin moves inward, and then cells are finally rounded up and prepared for division [29]. This indicates that cell-matrix detachment could be a consequence of rapid cell division. Therefore, we inhibited cell proliferation using aphidicolin or genistein and found that claudin-7 KD cells partially improved the cell attachment measured by the prolonged trypsinization time although KD cells still took less time to be lifted from the plates than the control cells (data not shown). However, we did not detect the increased level of integrin β1 after drug treatment. Therefore, the improved cell attachment could be due to the increased cell size with the larger attachment surface.

Type IV collagen is the structural backbone of the basement membrane of several solid organs including lung and has the ability to interact with the cell surface adhesion molecules such as integrins. Our results show that several collagens were significantly decreased at mRNA levels in claudin-7 KD cells. Culturing claudin-7 KD cells on collagen IV coated-plates partially reversed the morphology of claudin-7 KD cells in that they became larger in size, more spread out, and had improved cell attachment. However, cell number count revealed that claudin-7 KD cells still displayed a higher proliferation rate than the control cells when culturing on type IV collagen-coated plate. Type IV collagen has been noted to have disparate effects on adhesion and proliferation in other cell lines. Adhesion of melanoma cells to type IV collagen is enhanced by 50 %, but proliferation is inhibited by 40 % when compared to controls [30]. The proliferation of rat cortical progenitor cells is also inhibited on type IV collagen [31]. In the case of hepatoma cells, both adhesion and proliferation increase on type IV collagen [32]. Our results suggest that cell attachment depends on both cell proliferation and integrin β1 level, whereas cell proliferation does not depend on cell attachment. Both enhanced cell proliferation rate and decreased integrin β1 level are the primary results of claudin-7 suppression in HCC827 and H358 lung cancer cells.

To conclude, in this study, we have discovered a novel function of claudin-7 as a basolateral protein in regulating cell proliferation and cell-matrix adhesion in lung cancer cells. This study extends our current understanding of the roles of claudins in carcinogenesis. Both claudins and integrins play essential regulatory roles in tumor proliferation, invasion and metastasis. Understanding the molecular mechanisms of the lung cancer cell-matrix adhesions could lead to the identification of novel therapeutic strategies to target tumor cells. This novel basolateral function of claudin-7 engaging integrin β1 in human lung cancer cells could provide a previously unidentified therapeutic target in the future.

## Conclusion

Our study suggests a tumor suppression role of claudin-7 in lung cancer growth and identifies a new function of claudin-7 in maintaining epithelial cell attachment through interaction with integrin β1.

## Materials and methods

### Antibodies and reagents

The rabbit polyclonal anti-claudin-1 (Cat. 51–9000), −2 (Cat. 516100), −3 (Cat. 341700), −4 (Cat. 364800), ZO-1 (Cat. 61–7300), and mouse monoclonal anti-myc (Cat. 46–0603) antibodies were from Invitrogen (Carlsbad, CA). The rabbit polyclonal anti-claudin-7 antibody (Cat. 18875) was obtained from Immuno-Biological Laboratories (Japan). The anti-E-cadherin antibody (Cat. 610182, clone: 36) was purchased from BD Biosciences (San Jose, CA). The rabbit polyclonal anti-phospho-Bcl-2 (Cat. A7025, Ser56) was from Assay Biotechnology (San Francisco, CA). The rabbit polyclonal anti-phospho-FAK (Cat. 3283S) and anti-phospho-ERK1/2 (Cat. 9101S) were from Cell Signaling Technology (Beverly, MA). The mouse monoclonal and the goat polyclonal anti-integrin β1 antibodies were purchased from BD Biosciences (Cat. 610467) and Santa Cruz Biotechnology (Cat. sc-6622, Santa Cruz, CA), respectively. The mouse anti-integrin β1 adhesion-blocking antibody was from Chemicon (Cat. MAB22253Z, Millipore, MA).

### Cell culture, lentivirus shRNA knockdown of claudin-7, transfection of claudin-7 tagged with myc

Lung adenocarcinoma cell line HCC827, lung carcinoma cell line NCI-H358 (H358) and mammary ductal carcinoma cell line T-47D were purchased from ATCC (Manassas, VA) and grown in RPMI 1640 culture medium containing 10 % FBS, 100 units/ml of penicillin, and 100 μg/ml streptomycin in a humidified air (5 % CO_2_ atmosphere) at 37 °C. Each of three lentivirus claudin-7 shRNA vectors (sequences #1: 5′-TTCCAAGGAGTATGTGTGA-3′; #2: 5′-GGCTATGGGAGTGTCTAGA-3′; #3: 5′-TCCCTACCAACATTAAGTA-3′) or one off-target shRNA control vector were transfected into HCC827, H358 or T-47D cells. The cells infected with #2 lentivirus claudin-7 shRNA vector are designated as KD cells, and the cells infected with #3 shRNA vector are designated as KD2 cells. All lentivirus vectors contain a GFP expression sequence. After 48 h incubation, transfected cells were selected by 1 μg/ml puromycin.

The pcDNA3.1 myc or pcDNA3.1-*claudin-7*-myc (mouse *claudin-7* cDNA) was transfected into HCC827 claudin-7 KD cells using Amaxa Nucleofector device (Amaxa Biosystems, Cologne, Germany). Geneticin (G418) was added to select the transfected cells.

### Cell number count and cell cycle analysis by flow cytometry

Five × 10^3^ HCC827 or H358 control or claudin-7 KD cells were seeded into each well of 24-well culture plates and then trypsinized on days 2, 4, 6 after plating, respectively. One × 10^5^ 6 micron AlignFlow Plus beads (Molecular Probes, Eugene, OR) were added to each sample and relative ratio of beads versus cells was obtained by the flow cytometer. The total cell number for each sample was then calculated.

HCC827 control or claudin-7 KD cells untreated or treated with 5 μg/ml aphidicolin for 24 h were harvested, washed with PBS, and fixed in 70 % ethanol. Prior to analysis, cells were washed with PBS and then resuspended in the propidium iodide staining solution. Samples were analyzed on the flow cytometer.

### RNA isolation and real-time RT-PCR

Total RNA was isolated from HCC827 control and claudin-7 KD cells using Qiagen RNeasy Kit (Qiagen, Valencia, CA) following the manufacturer’s instructions and reverse transcripted into cDNA using RT^2^ First Strand Kit (Qiagen). Real-time RT-PCR was performed using the RT^2^ SYBR Green qPCR Mix (Qiagen). The relative changes in gene expression were analyzed using 2^-ΔΔCt^ method [16].

### Immunofluorescence

HCC827 or H358 control or claudin-7 KD cells grown on poly-D-lysine coated glass coverslips (BD Biosciences, Bedford MA) were fixed in 100 % methanol for 8 min at −20 °C and washed with PBS for 5 min before blocked in 5 % bovine serum albumin for 60 min at room temperature. After blocking, cells were incubated with primary antibodies. All antibodies were diluted in PBS containing 2.5 % BSA. After washing, cells were incubated with corresponding secondary antibody for 45 min at room temperature. Coverslips were mounted with ProLong Antifade Kit (Molecular Probes Inc., OR). Samples were photographed using a Zeiss Axiovert S100 or Zeiss LSM 510 laser confocal scanning microscopy (Carl Zeiss Inc., Thornwood, NY) and analyzed by MetaMorph software (Molecular Devices, Sunnyvale, CA).

### Co-immunoprecipitation

HCC827 or H358 control cells were washed three times with ice-cold PBS and then lysed in RIPA buffer. After centrifugation, the supernatants were incubated with either anti-claudin-7 or anti-integrin β1 antibody at 4 °C overnight. Protein A or protein G beads were then added to the mixture, followed by incubation at 4 °C for 3 h. The beads were washed twice with RIPA buffer, once with high salt buffer (0.5 M NaCl), and once with Tris buffer (10 mM Tris, pH 7.4). Bound proteins were eluted from the beads in SDS sample buffer and analyzed by western blot.

### Measurement of transepithelial electrical resistance (TER)

For TER measurement, HCC827 control or claudin-7 KD cells were plated on collagen-coated Transwell filters with a pore size of 0.4 μm (Corning Costar, Cambridge, MA). A millicell-ERS volt-ohm meter (Millipore, Bedford, MA) was used to determine the TER value. All the TER values were normalized for the area of the filter and obtained after background subtraction.

### *In vivo* tumor xenograft model

Five-week old male athymic nude mice were obtained from Charles River Laboratory (Wilmington, MA) and used for human tumor xenografts. Two × 10^6^ HCC827 control or claudin-7 KD cells were suspended in the culture medium and injected subcutaneously into the left and right flanks of each nude mouse, respectively. All the mice were sacrificed eight weeks after injection. The tumors were removed and weighed. The animal experiments were performed according to the animal use protocol (AUP) approved by East Carolina University.

### Statistical analysis

Statistical analysis was performed using Origin50 (OriginLab, MA) or SigmaPlot (SPSS Science, Chicago, IL) software. For each *in vitro* experiment, at least three independent experiments were performed. All data was recorded and expressed as means ± SE. The differences between two groups were analyzed using the unpaired Student’s *t*-test. The data from the nude mice tumor xnenograft experiment was analyzed using Wilcoxon Rank-Sum Test. All statistical tests were two-sided and a value of *P* < 0.05 was considered significant (*).

## Additional files

Additional file 1: Figure S1. Knockdown of claudin-7 using #3 shRNA lentivirus construct against claudin-7 (CLDN7 KD2) in HCC827 lung cancer cells. (A) Immunofluorescence images of control and claudin-7 KD2 cells using anti-claudin-7 antibody. The control and KD2 cells were grown on coverslips and fixed by 100 % methanol. Claudin-7 signal was greatly reduced in claudin-7 KD2 cells. Bar: 10 μm. (B) Representative Western blot results show the decreased expression level of claudin-7 in the KD2 cells compared to that of the control cells. Additional file 2: Figure S2. Knockdown of claudin-7 using #2 shRNA lentivirus construct against claudin-7 in H358 lung cancer cells. (A) Immunofluorescence images of H358 control and claudin-7 KD cells using anti-claudin-7 antibody. The cells were fixed with 100 % methanol. Bar: 20 μm. (B) Western blotting shows that the expression level of claudin-7 is greatly reduced in the KD cells compared to that of the control cells. Additional file 3: Figure S3. Increased cell proliferation in HCC827 claudin-7 KD2 cells. (A) Five × 10^3^ control and claudin-7 KD2 cells were seeded into 24-well plates, and then the cell number was counted on days 2, 4, and 6 after each sample was plated. Claudin-7 KD2 cells showed a significantly higher proliferation rate compared to the control cells on all three days tested. **P* < 0.05. (B) Representative Western blots show an increased level of phospho-ERK1/2 and a decreased level of cleaved PARP in claudin-7 KD2 cells when compared to those of the control cells. Additional file 4: Figure S4. Increased cell proliferation in H358 claudin-7 KD cells. (A) Five × 10^3^ H358 control and claudin-7 KD cells were seeded into 24-well plates. The cell number was counted on 2, 4, and 6 days after the cells were plated. Claudin-7 KD cells displayed a significantly higher proliferation rate compared to the control cells on days 4 and 6. **P* < 0.05. (B) Representative Western blots show an increased level of phospho-ERK1/2 and a decreased level of cleaved PARP in claudin-7 KD cells when compared to those of the control cells while the total ERK1/2 was unchanged. Additional file 5: Figure S5. Reduced cell apoptosis in HCC827 claudin-7 KD cells. (A). HCC827 control and claudin-7 KD cells were fixed by 100 % methanol and incubated with 10 % BSA in PBS for 30 min at 37 °C before applying TUNEL reaction mixture (Roche Diagnostics, Indianapolis, IN, Cat. 12156792910) to the cells for one hour at 37 °C. The red signal indicates the apoptotic cells. The blue signal is the nuclear staining. Bar: 50 μm. (B) The percentage of cell apoptosis was significantly lower in HCC827 claudin-7 KD cells compared to that of the control cells. Data was analyzed from five different samples. **P* < 0.05. Additional file 6: Figure S6. Reduced cell attachment ability in HCC827 claudin-7 KD2 cells. (A) Cell attachment assay. Two × 10^5^ control and KD2 cells were plated to each well of the collagen IV-coated 24-well plates. Four hours later, the unattached cells were washed off and the attached cells were trypsinized and counted. KD2 cells showed significantly less cell attachment compared to the control cells. **P* < 0.05. (B) Western blots showed that integrin β1 and phospho-FAK levels were decreased in claudin-7 KD2 cells. At least three independent experiments were performed. Additional file 7: Figure S7. Single immunofluorescent staining of integrin β1 and claudin-7 on HCC827 cells. (A) HCC827 control cells were grown on the poly-D-lysine coated glass coverslips and then fixed in 100 % methanol for 8 min at −20 °C. After blocking, cells were incubated with mouse anti-integrin β1 antibody for one hour at room temperature. Coverslips were mounted with ProLong Antifade Kit and samples were photographed using a Zeiss Axiovert S100. Both low density (top) and high density (bottom) cells were shown. Bar: 15 μm. (B) HCC827 control cells were treated the same as in (A) except that the primary antibody was the rabbit anti-claudin-7 antibody. The secondary antibodies were Cy3 (for integrin β1) and FITC (for claudin-7), respectively. Additional file 8: Figure S8. The cell attachment defect in H358 claudin-7 KD cells. (A) When cultured on uncoated glass coverslips, H358 claudin-7 KD cells formed spheroids on both 2-day (2d) and 5-day (5d) cultures while the control cells were able to spread out and form a monolayer. Bar: 30 μm. (B) Two × 10^5^ H358 control and KD cells were plated to each well of 24-well plates. Four hours later, the unattached cells were washed off and the attached cells were trypsinized and counted. Claudin-7 KD cells showed significantly reduced cell attachment compared to that of the control cells. **P* < 0.05. Additional file 9: Figure S9. Reduced integrin β1 expression level in H358 claudin-7 KD cells. (A) Western blots show that integrin β1 and phospho-FAK levels were decreased in H358 claudin-7 KD cells compared to the control (CON) cells. (B) Double immunofluorescence staining of claudin-7 and integrin β1 in H358 control cells. Arrows in control cells indicate the partial co-localization of claudin-7 with integrin β1. (C) Claudin-7 co-immunoprecipitated with integrin β1. Control cells were lysed in RIPA buffer without SDS and immunoprecipitated with either anti-integrin β1 or anti-claudin-7 antibody. The membrane was probed with either claudin-7 or integrin β1.
