# Supplementary figures and images for: A non-tight junction function of claudin-7—Interaction with integrin signaling in suppressing lung cancer cell proliferation and detachment
Source: Mol Cancer. 2015 Jun 17;14:120. doi: 10.1186/s12943-015-0387-0 (PMC4470020; doi:10.1186/s12943-015-0387-0)

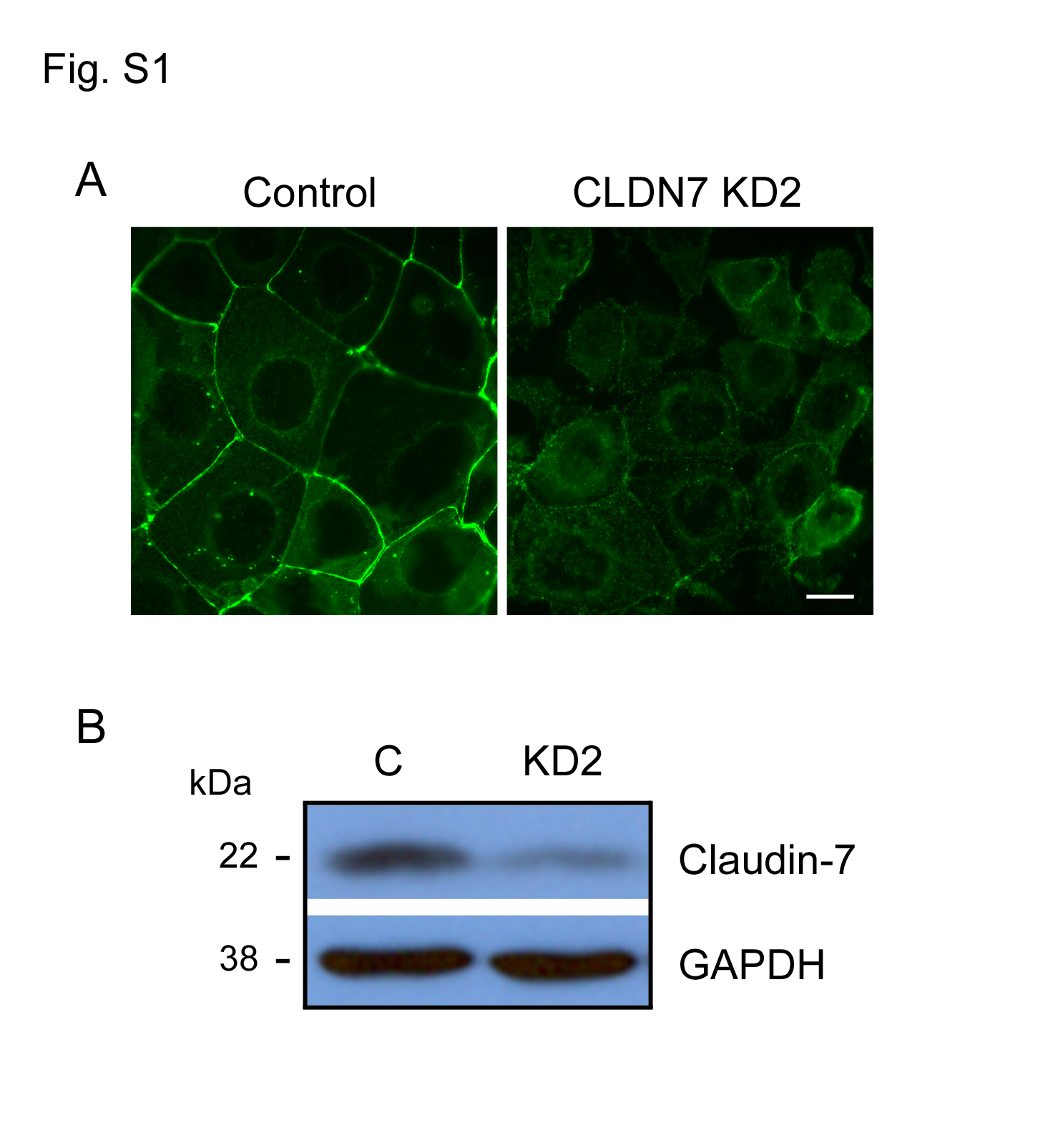

Supplement: Additional file 1: Figure S1. — Knockdown of claudin-7 using #3 shRNA lentivirus construct against claudin-7 (CLDN7 KD2) in HCC827 lung cancer cells. (A) Immunofluorescence images of control and claudin-7 KD2 cells using anti-claudin-7 antibody. The control and KD2 cells were grown on coverslips and fixed by 100 % methanol. Claudin-7 signal was greatly reduced in claudin-7 KD2 cells. Bar: 10 μm. (B) Representative Western blot results show the decreased expression level of claudin-7 in the KD2 cells compared to that of the control cells. [file 12943_2015_387_MOESM1_ESM.tiff]

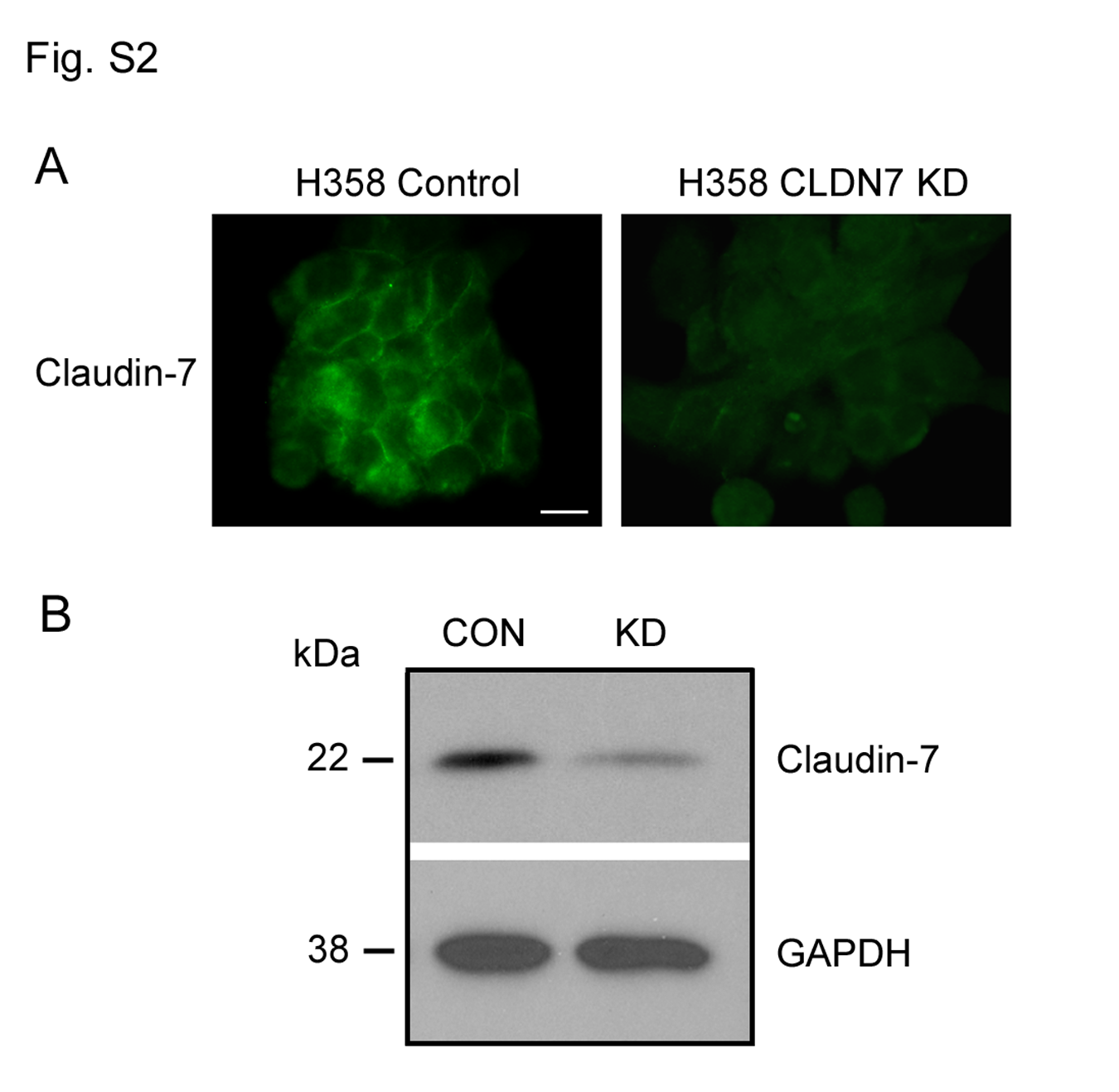

Supplement: Additional file 2: Figure S2. — Knockdown of claudin-7 using #2 shRNA lentivirus construct against claudin-7 in H358 lung cancer cells. (A) Immunofluorescence images of H358 control and claudin-7 KD cells using anti-claudin-7 antibody. The cells were fixed with 100 % methanol. Bar: 20 μm. (B) Western blotting shows that the expression level of claudin-7 is greatly reduced in the KD cells compared to that of the control cells. [file 12943_2015_387_MOESM2_ESM.tiff]

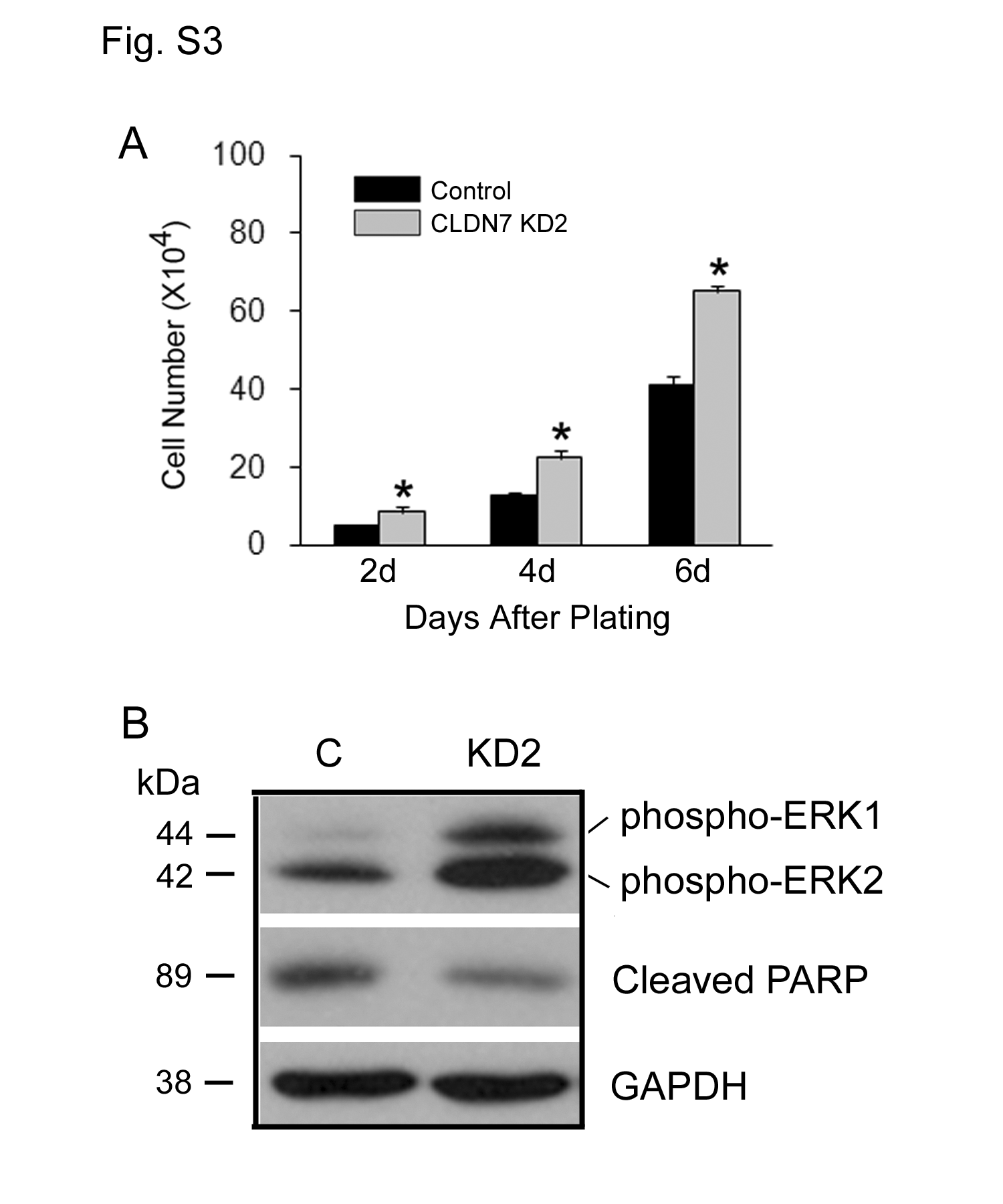

Supplement: Additional file 3: Figure S3. — Increased cell proliferation in HCC827 claudin-7 KD2 cells. (A) Five × 103 control and claudin-7 KD2 cells were seeded into 24-well plates, and then the cell number was counted on days 2, 4, and 6 after each sample was plated. Claudin-7 KD2 cells showed a significantly higher proliferation rate compared to the control cells on all three days tested. *P < 0.05. (B) Representative Western blots show an increased level of phospho-ERK1/2 and a decreased level of cleaved PARP in claudin-7 KD2 cells when compared to those of the control cells. [file 12943_2015_387_MOESM3_ESM.tiff]

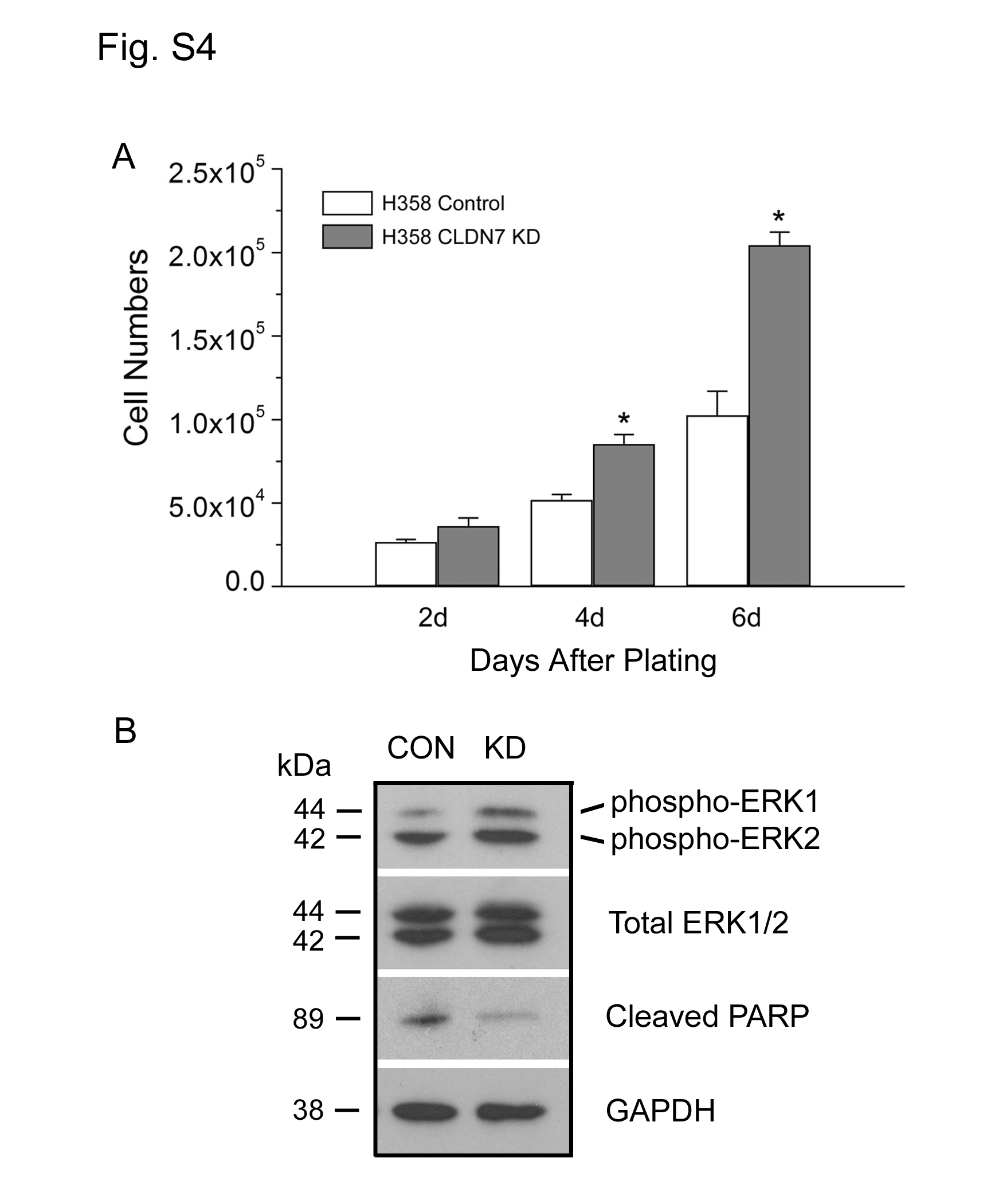

Supplement: Additional file 4: Figure S4. — Increased cell proliferation in H358 claudin-7 KD cells. (A) Five × 103 H358 control and claudin-7 KD cells were seeded into 24-well plates. The cell number was counted on 2, 4, and 6 days after the cells were plated. Claudin-7 KD cells displayed a significantly higher proliferation rate compared to the control cells on days 4 and 6. *P < 0.05. (B) Representative Western blots show an increased level of phospho-ERK1/2 and a decreased level of cleaved PARP in claudin-7 KD cells when compared to those of the control cells while the total ERK1/2 was unchanged. [file 12943_2015_387_MOESM4_ESM.tiff]

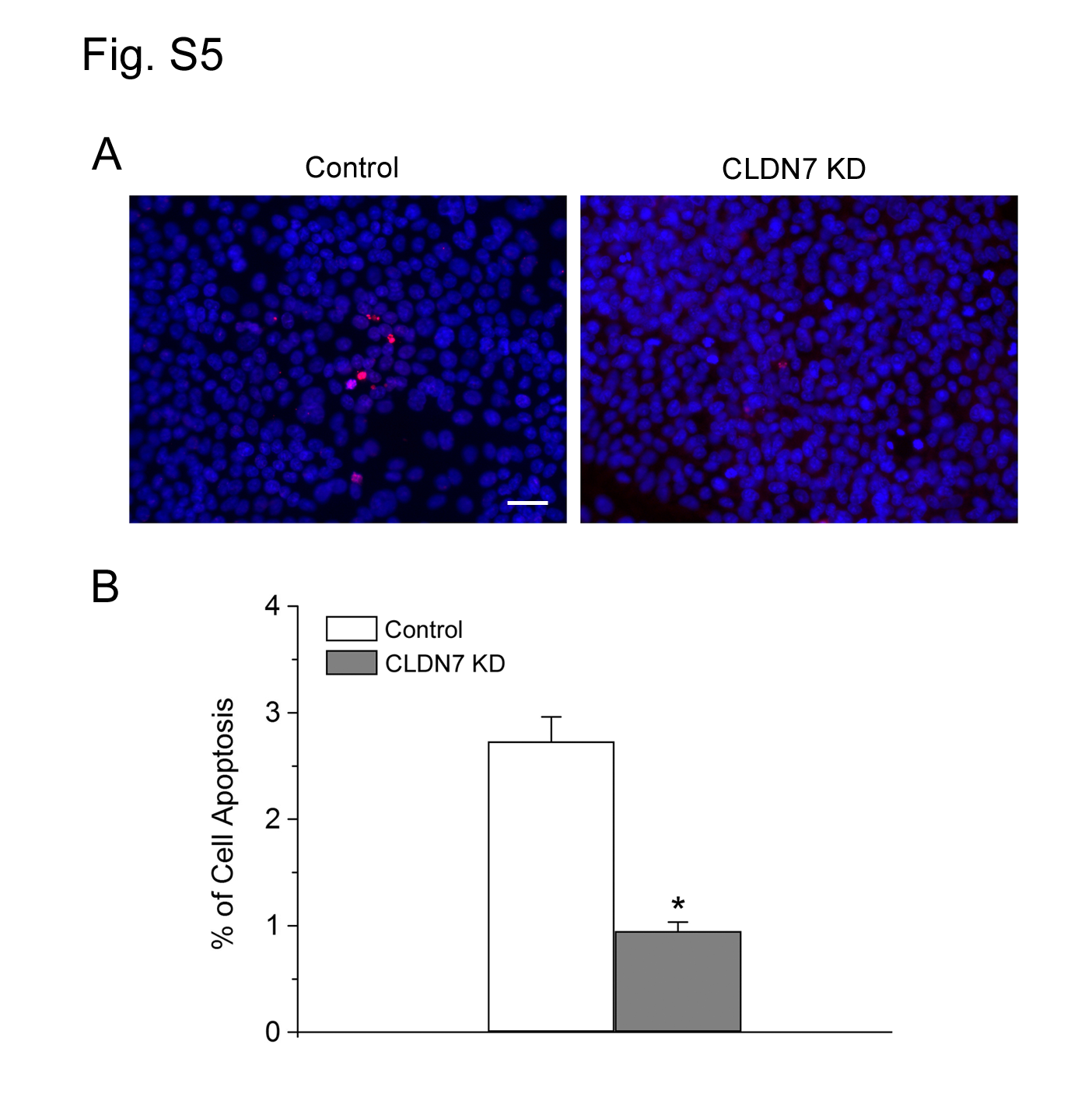

Supplement: Additional file 5: Figure S5. — Reduced cell apoptosis in HCC827 claudin-7 KD cells. (A). HCC827 control and claudin-7 KD cells were fixed by 100 % methanol and incubated with 10 % BSA in PBS for 30 min at 37 °C before applying TUNEL reaction mixture (Roche Diagnostics, Indianapolis, IN, Cat. 12156792910) to the cells for one hour at 37 °C. The red signal indicates the apoptotic cells. The blue signal is the nuclear staining. Bar: 50 μm. (B) The percentage of cell apoptosis was significantly lower in HCC827 claudin-7 KD cells compared to that of the control cells. Data was analyzed from five different samples. *P < 0.05. [file 12943_2015_387_MOESM5_ESM.tiff]

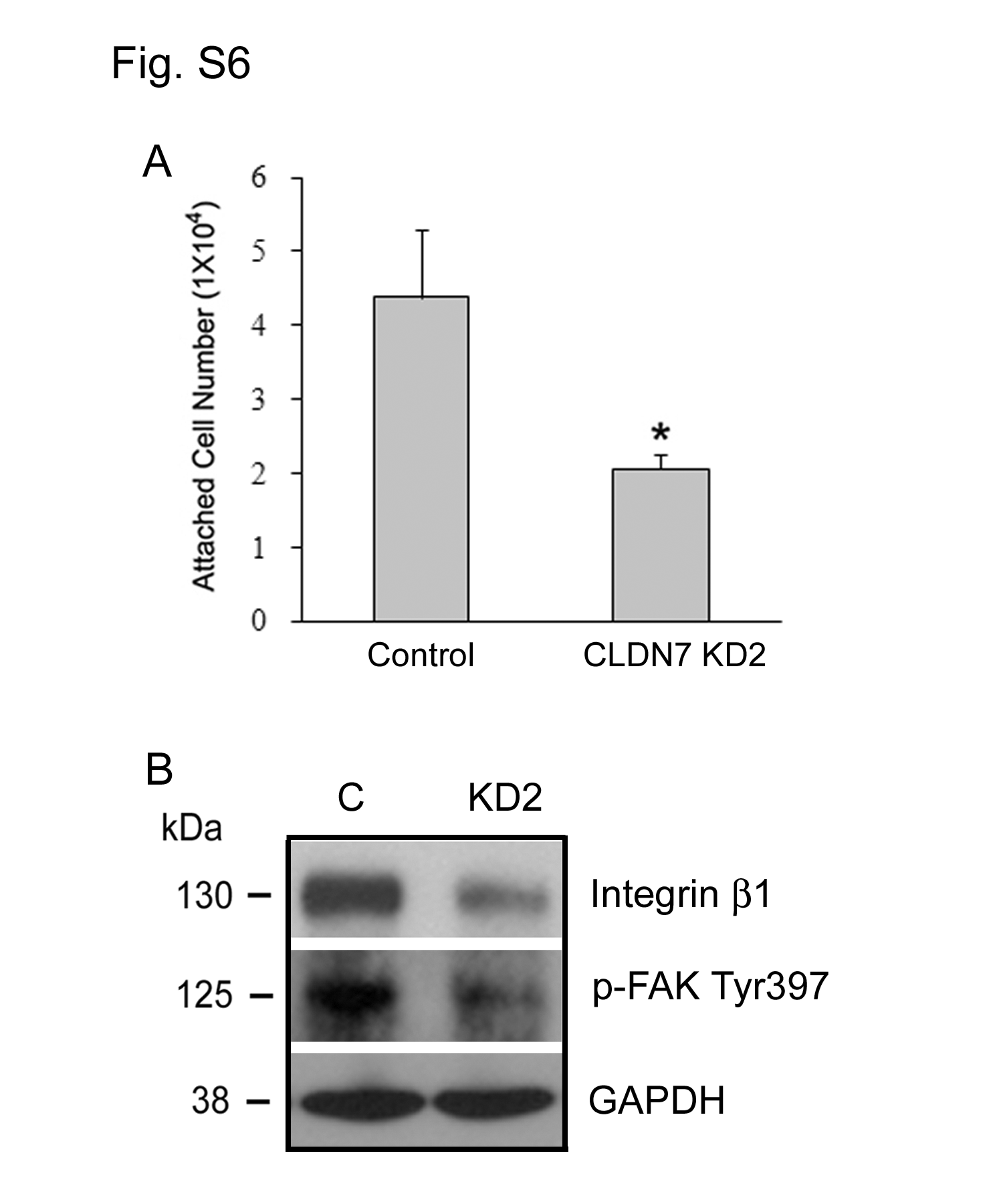

Supplement: Additional file 6: Figure S6. — Reduced cell attachment ability in HCC827 claudin-7 KD2 cells. (A) Cell attachment assay. Two × 105 control and KD2 cells were plated to each well of the collagen IV-coated 24-well plates. Four hours later, the unattached cells were washed off and the attached cells were trypsinized and counted. KD2 cells showed significantly less cell attachment compared to the control cells. *P < 0.05. (B) Western blots showed that integrin β1 and phospho-FAK levels were decreased in claudin-7 KD2 cells. At least three independent experiments were performed. [file 12943_2015_387_MOESM6_ESM.tiff]

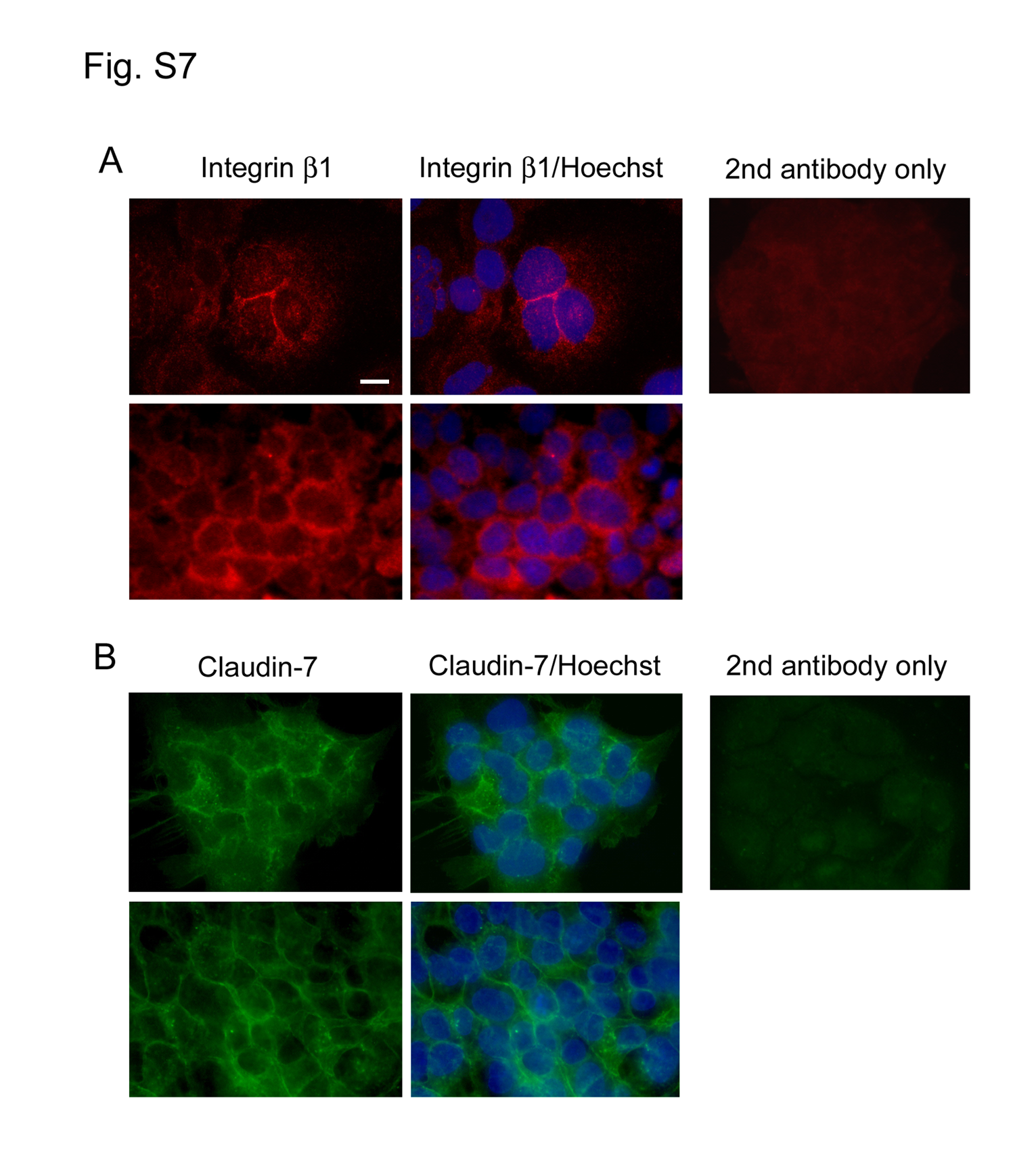

Supplement: Additional file 7: Figure S7. — Single immunofluorescent staining of integrin β1 and claudin-7 on HCC827 cells. (A) HCC827 control cells were grown on the poly-D-lysine coated glass coverslips and then fixed in 100 % methanol for 8 min at −20 °C. After blocking, cells were incubated with mouse anti-integrin β1 antibody for one hour at room temperature. Coverslips were mounted with ProLong Antifade Kit and samples were photographed using a Zeiss Axiovert S100. Both low density (top) and high density (bottom) cells were shown. Bar: 15 μm. (B) HCC827 control cells were treated the same as in (A) except that the primary antibody was the rabbit anti-claudin-7 antibody. The secondary antibodies were Cy3 (for integrin β1) and FITC (for claudin-7), respectively. [file 12943_2015_387_MOESM7_ESM.tiff]

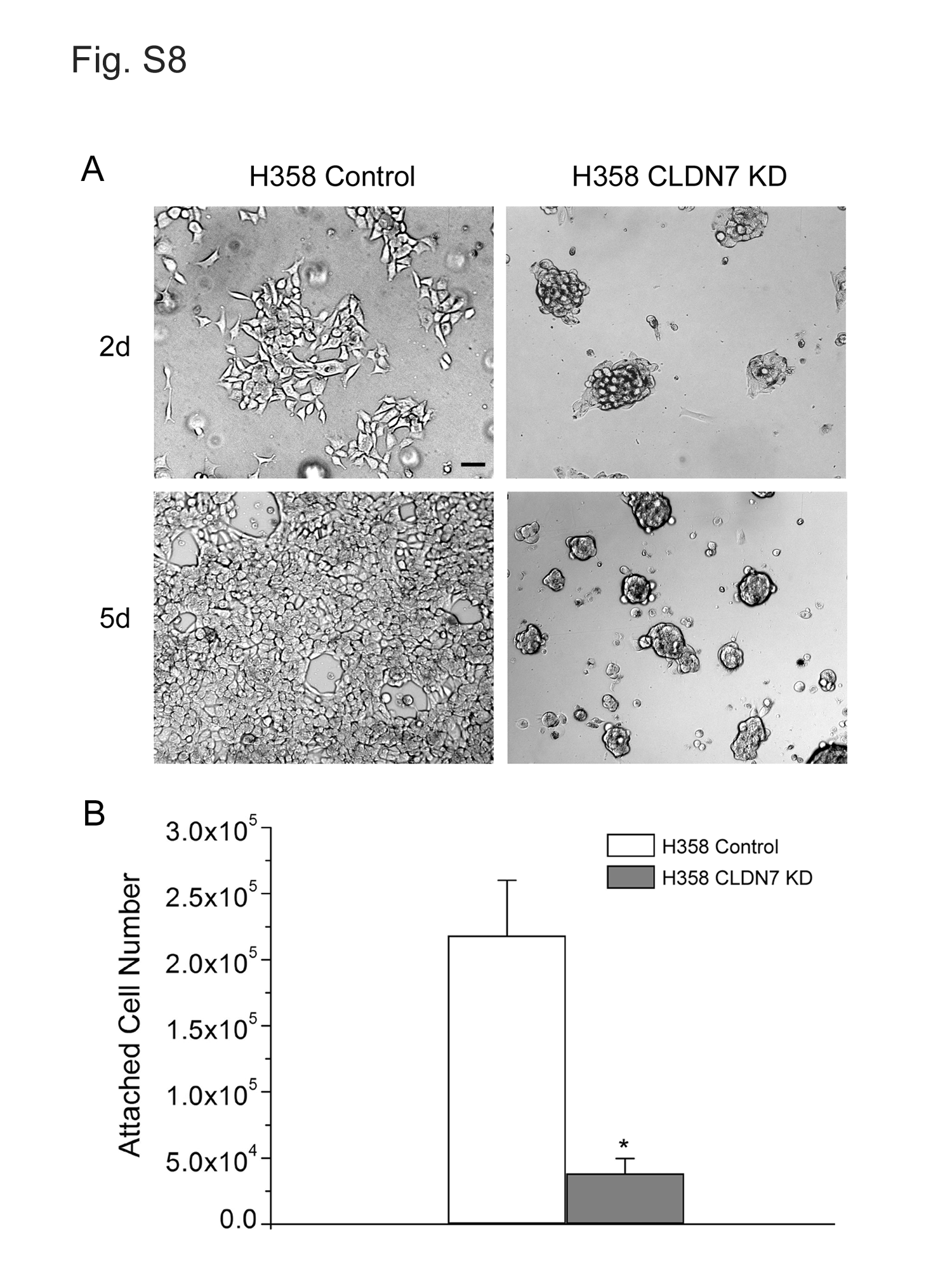

Supplement: Additional file 8: Figure S8. — The cell attachment defect in H358 claudin-7 KD cells. (A) When cultured on uncoated glass coverslips, H358 claudin-7 KD cells formed spheroids on both 2-day (2d) and 5-day (5d) cultures while the control cells were able to spread out and form a monolayer. Bar: 30 μm. (B) Two × 105 H358 control and KD cells were plated to each well of 24-well plates. Four hours later, the unattached cells were washed off and the attached cells were trypsinized and counted. Claudin-7 KD cells showed significantly reduced cell attachment compared to that of the control cells. *P < 0.05. [file 12943_2015_387_MOESM8_ESM.tiff]

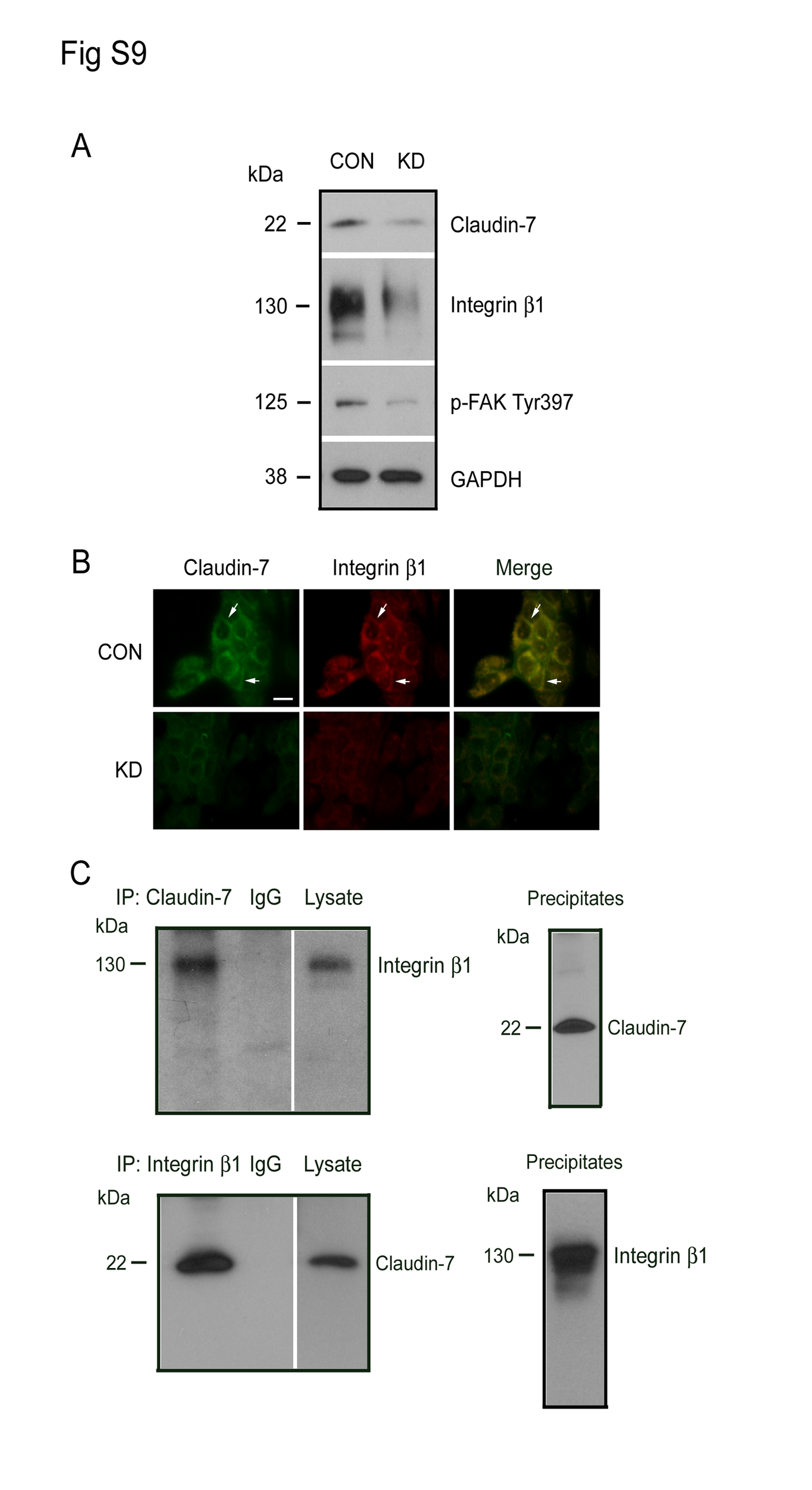

Supplement: Additional file 9: Figure S9. — Reduced integrin β1 expression level in H358 claudin-7 KD cells. (A) Western blots show that integrin β1 and phospho-FAK levels were decreased in H358 claudin-7 KD cells compared to the control (CON) cells. (B) Double immunofluorescence staining of claudin-7 and integrin β1 in H358 control cells. Arrows in control cells indicate the partial co-localization of claudin-7 with integrin β1. (C) Claudin-7 co-immunoprecipitated with integrin β1. Control cells were lysed in RIPA buffer without SDS and immunoprecipitated with either anti-integrin β1 or anti-claudin-7 antibody. The membrane was probed with either claudin-7 or integrin β1. [file 12943_2015_387_MOESM9_ESM.tiff]
